# 

**Published:** 1890-03

**Authors:** 


CONTENTS:

PAGE

ORIGINAL ARTICLES.—
- Contagious Venereal Disease Amongst Horses in Kent County, Canada. By Peter H. Bryce, M.A., M D. . . . . 143
- On the Influence of Slight Modifications of Culture Media on the Growth of Bacteria as Illustrated by the Glanders Bacillus. By Theobald Smith, M.D. . . . . 158
- The Present Attitude of Veterinarians on the Subject of Tuberculosis. By Daniel Lee, M.D.V. . . . . 162
- Entropion in a Dog. (Dianour's Operation.) By Gerald G. Griffin, D.V.S. 164
- Heart Disease in the Horse. By John A. McLaughlin, D.V.S. . . . . 165
- Age of the Horse, Ox, Dog, and other Domestic Animals. By R. S. Huidekoper, M.D., Veterinarian . . . . 167
- Gastrotomy. By E. S. Lawley . . . . 173

EDITORIAL DEPARTMENT.—
- Veterinary Progress a Gain to Science . . . . 175
- United States Veterinary Medical Association . . . . 178

CASES, SELECTIONS, TRANSLATIONS.—
- Pathology in Its Relations to General Biology. By William H. Welch, M.D. 179
- Contagious Pleuro-pneumonia in Goats at Cape Colony, South Africa . . 183
- Foreign Bodies Removed from the Eye . . . . 187

SOCIETY PROCEEDINGS . . . . 188
VETERINARY COLLEGE NOTES . . . . 194
PERSONAL . . . . 194
REVIEW DEPARTMENT . . . . 195
BOOKS AND PAMPHLETS RECEIVED . . . . 195

